# Integrated Network Pharmacology and Metabolomics Analysis of the Therapeutic Effects of Zi Dian Fang on Immune Thrombocytopenic Purpura

**DOI:** 10.3389/fphar.2018.00597

**Published:** 2018-06-19

**Authors:** Yubo Li, Yamei Li, Wenliang Lu, Hongbin Li, Yuming Wang, Houmin Luo, Yuanyuan Wu, Wenying Dong, Gang Bai, Yanjun Zhang

**Affiliations:** ^1^Tianjin State Key Laboratory of Modern Chinese Medicine, School of Traditional Chinese Materia Medica, Tianjin University of Traditional Chinese Medicine, Tianjin, China; ^2^Tasly Institute, Tasly Pharmaceutical Group, Tianjin, China; ^3^College of Pharmacy, Nankai University, Tianjin, China

**Keywords:** network pharmacology, metabonomics, biomarkers, immune thrombocytopenic purpura, mechanism

## Abstract

Current hormone-based treatments for immune thrombocytopenic purpura (ITP) are associated with potentially serious adverse reactions. Zi Dian Fang (ZDF) is a multi-target Traditional Chinese Medicine (TCM) used to treat both the symptoms and root causes of ITP, with fewer side effects than hormone-based treatments. This study analysis of the therapeutic effects of ZDF on ITP from three aspects: platelet proliferation, immunoregulation, and inflammation. After detection of 52 chemical constituents of ZDF by UPLC-Q-TOF/MS, The main targets and pathways affected by ZDF were screened by network pharmacology and verified by Western blot and ELISA. Meanwhile, metabolomics analysis were applied to a mouse model of ITP to identify and screen endogenous terminal metabolites differentially regulated by ZDF. Integrated network pharmacology and metabolomics analysis of the therapeutic effects of ZDF on ITP may be as follows: ZDF counteracts ITP symptoms mainly by inhibiting Ras/MAPKs (Ras/Mitogen-activated protein kinases) pathway, and the expression of upstream protein (Ras) and downstream protein (p-ERK, p-JNK, p-p38) were inhibited, which affects the content of effect index associated with proliferation (Thrombopoietin, TPO; Granulocyte-macrophage colony stimulating factor, GM-CSF), inflammation (Tumor necrosis factor-α, TNF-α; Interleukin-6, IL-6), immune (Interleukin-2, IL-2; Interferon-gamma, IFN-γ; Interleukin-4, IL-4), so that the body’s arginine, Δ^12^-prostaglandin j2 (Δ^12^-PGJ2), 9-*cis*-Retinoic Acid, sphingosine-1-phosphate (S1P), oleic acid amide and other 12 endogenous metabolites significantly changes. Considering the established safety profile, the present study suggests ZDF may be a useful alternative to hormone-based therapies for the treatment of ITP.

## Introduction

Immune thrombocytopenic purpura (ITP) is an autoimmune vasculitis characterized by skin, mucosal and/or visceral bleeding resulting from autoantibody-mediated platelet damage (Rodeghiero et al., 2009). ITP is a relatively common hemorrhagic disease in children. While acute ITP typically resolves within 3 months, chronic refractory ITP has characteristic long disease course (Swain et al., 2016; Queliza et al., 2017). Adrenocortical hormone therapy (prednisone acetate, cortisone, etc.) is often used for the clinical treatment of ITP, but is associated with adverse reactions, and long-term use seriously compromises the body’s defense system (Lembke et al., 2013; Arnett et al., 2016). Traditional Chinese Medicine (TCM) offers a holistic approach to patient treatment and care, viewing disease as the result of an internal imbalance between the body’s energy (Yang) and substance (Yin). Because TCM aims to restore homeostasis basically by normalizing immune and metabolic functions, multi-ingredient TCM formulae are particularly suited for the treatment of complex diseases. Naturally sourced TCM preparations have been used, and their safety tested, over 1000s of years in East Asian countries. However, lack of characterization of the biologically active principles in TCM herbal formulations and lack of evidence-based standards of quality and efficacy have hampered their acceptance and use in other countries (Zhou et al., 2014).

Zi Dian Fang (ZDF) is a TCM formula composed of *Hedysarum multijugum Maxim* (zhihuangqi in Chinese, ZHQ), *Cortex Moutan* (mudanpi in Chinese, MDP), *Fructus Ligustri Lucidi* (nvzhenzi in Chinese, NZZ), *Ecliptae Herba* (mohanlian in Chinese, MHL), *Sweet potato vine* (fanshuteng, FST), *Donkey-hide gelatin* (ejiao in Chinese, AJ), *Herba Selaginellae Moellendorffii* (juanbai in Chinese, JB), *Panax notoginseng* (sanqi in Chinese, SQ), *Peanut skin* (huashengyi in Chinese, HSY), and *Radix liquiritiae* (gancao in Chinese, GC). According to TCM precepts, this herbal decoction is used to treat ITP by ‘benefiting *qi*’ (vital energy/flow) and ‘nourishing *yin*,’ i.e., ‘cooling the blood’ and favoring hemostasis. However, its chemical composition and the molecular basis of its therapeutic effects on ITP have not been fully studied (Han et al., 2014; Wagner et al., 2016).

Complex diseases are often caused by the accumulation of small defects in many genes, rather than large defects in a few ones; therefore, treatments aimed at single molecular targets frequently do not work (Falvo et al., 2013). In 2007, Andrew L. Hopkins introduced the concept of “Network pharmacology,” its core idea being to identify the biological networks that interconnect disease features, bioactive agents, and drug targets with the goal of optimizing treatment strategies. Interestingly, the core idea of network pharmacology coincides with the tenet of overall regulation of TCM (Hopkins, 2008). Thus, network pharmacology analysis has been applied to the study of TCM formulae to predict the molecular targets and signaling pathways affected by TCM prescriptions for different diseases (Chen et al., 2015; Mao et al., 2017). Prescription compatibility, which refers to the structural composition of herbal medicines, is flexible and complex, as elimination of the original substance, generation of new substances, or changes in the contents of active ingredients may occur in the process of boiling and extraction. However, network pharmacology can only be used to forecast targets and pathways affected by individual chemical components; it can not explain changes in metabolites that occur in the body after taking the medicine, so it requires cellular and/or animal experimental verification and further support of ‘omics’ technologies such as chemomics and metabolomics (Pang et al., 2018).

The metabolomics technology emerges with the development of systems biology, and is an important part of it. It takes the whole organism as the research object, studies the effects of drugs and other factors on terminal metabolites by observing the changes that result after stimulating or disturbing the biological system, and then explains drugs actions at the metabolic level (Shajahan-Haq et al., 2015; Ju et al., 2016). Thus, the combination of network pharmacology and metabolomics allows identifying and connecting biologically active substances, molecular targets, and metabolic effects and is thus ideally suited to study the mechanistic basis of TCM treatments.

In this study, we first identified the chemical constituents of ZDF by UPLC-Q-TOF/MS, a highly sensitive and specific technique (Mejías et al., 2014). Next, we applied network pharmacology methods to generate an ITP “chemical composition-target-pathway” regulatory network centered on three main aspects: platelet proliferation, immunity, and inflammation. Subsequently, we used an ITP mouse model and metabolomics analysis to verify ZDF-induced alterations in the major predicted pathway, i.e., the Ras/MAPKs signaling pathway, which includes Ras and its main downstream targets: ERK, JNK, and p38MAPK. Finally, through integrative analysis of network pharmacology and metabolomics data we constructed a herb-chemical constituent-targets-pathway-regulatory index-metabolite regulatory network representation of ZDF’s pharmacological activity against ITP. By characterizing the ZDF composition and its therapeutic mechanisms on ITP we lay the foundation for the clinical application of this TCM prescription.

## Materials and Methods

### Instruments and Reagents

HPLC analysis was performed in a Waters UPLC-Q-TOF-MS system (Waters, United States). ACQUITY UPLC BEH C18 columns (2.1 × 100 mm, 1.7 μm, Waters), and ACQUITY UPLC HSS C18 columns (2.1 × 100 mm, 1.8 μm, Waters) were purchased from Waters, United States. Complete Freund’s adjuvant and incomplete Freund’s adjuvant were from Sigma, United States. Antibodies against p38, phospho-p38 (Thr 180/Tyr 182), p44/42 (ERK1/2), phospho-p44/42 (Thr 202/Tyr 204), SAPK/JNK, and phospho-SAPK/JNK (Thr 183/Tyr 185) were from Cell Signaling Technology, United States. Alkaline phosphatase-labeled goat anti-rabbit Ig G, RIPA lysis buffer, PMSF protease inhibitor, and protein phosphatase inhibitor mixture were purchased from Solarbio Co., Ltd. (Beijing, China). Mouse serum GM-CSF, TPO, IL-2, IL-4, IFN-γ, TNF-α, and IL-6 ELISA kits were purchased from Baoman Co., Ltd. (Shanghai, China). The BCA Protein Quantification Kit was purchased from Yupinghao Biological Technology Co., Ltd. (Tianjin, China). Purified water was purchased from Wahaha Company (Hangzhou, China). Prednisone acetate tablets were purchased from Lisheng Pharmaceutical Co., Ltd. (Tianjin, China). *Hedysarum multijugum Maxim, Cortex Moutan, Fructus Ligustri Lucidi, Ecliptae Herba, Sweet potato vine, Donkey-hide gelatin, Herba Selaginellae Moellendorffii, Panax notoginseng, Peanut skin, Radix liquiritiae* were purchased from Tong Ren Tang Co., Ltd. (Beijing, China).

### Preparation of Zi Dian Fang Test Solution

Ten medicinal herbs were used to prepare the ZDF test solution: ZHQ (25 g), MDP (10 g), NZZ (10 g), MHL (10 g), and SQ (3.35 g) were extracted three times with 8 times the amount of 70% ethanol under reflux for 60 min, following by filtering. FST (16.65 g), JB (10 g), HSY (5 g), and GC (5 g) were extracted three times with 8 times the amount of pure water under reflux for 60 min. Then the extracted solutions were filtered and combined with the alcohol extract. AJ (5 g) was mixed with the liquid medicine to prepare the ZDF test solution. Ten kinds of single medicinal herbs were extracted, collected and prepared according to the formula in accordance with the proportion of single herbs for the test solution.

### UPLC-Q-TOF/MS Analysis

#### Chromatographic Conditions

ACQUITY UPLC BEH C18 columns (2.1 × 100 mm, 1.7 μm, Waters); flow rate, 0.3 mL/min; column temperature, 30°C; injection volume, 5 μL. Mobile phases: A phase, 0.1% formic acid solution; B phase, acetonitrile. Specific elution procedures were as follows: 0–1 min, A: 99–99%; 1–10 min, A: 99–60%; 10–20 min, A: 60–30%; 30–31 min, A: 1–1%; 31–40 min, A: 1–99%.

#### Mass Spectrometry Conditions

Mass spectral analyses were carried out in positive ionization mode using ESI. Drying gas temperature, 350°C; drying gas flow, 10 mL/min; desolvation gas flow, 600 L/h; nebulizer pressure, 310 psi; range of data acquisition, 100–1500 Da.

### Data Analysis

Based on literature reports of herbs’ chemical composition, peak alignment and peak match lists were generated by MassLynx software, and the retention time, precise mass number, and secondary fragment information for each chemical component were obtained. Then, putative chemical categories and fragmentation rules of mass spectra were summarized. Finally, we compared the literature and standard information to confirm the chemical composition of ZDF. We obtained the total ion flow chart for single herbs, compared it with the total ion chromatogram of ZDF, and determined the ascription of each chemical component.

### Network Pharmacology Analysis

#### Construction of Zi Dian Fang “Chemical Constituent-Target-Pathway” Regulatory Network

Chemical constituents identified by UPLC-Q-TOF/MS were used as the research object. First, 3D structures were drawn using Chem Bio Office 2010 software and stored in sdf format. The Pharm Mapper server was used for potential target prediction analysis. The top 10 targets with high matching degrees were introduced into the Uni Prot database^1^ to obtain their official names for subsequent pathway analysis. Target information was imported into the MAS 3.0 database, and relevant pathway information was obtained. We analyzed the pathways using the gene analysis function stored in the KEGG database. Finally, Cytoscape 2.6.0 software was used to construct the “chemical composition-target-pathway” regulatory network of ZDF for ITP treatment.

#### Verification of Network Pharmacology-Based Virtual Screening Results

##### Animals

SPF-grade healthy BALB/c mice (8 weeks old, weighing 20 ± 2 g) were purchased from Beijing Weitong Lihua Technology Co. Ltd. under license number “SCXK (Jing) 2012-0001.” Ordinary level guinea pigs were purchased from Beijing Weitong Lihua Technology Co. Ltd. under license number “SCXK (Jing) 2012-0015.” The growth environment consisted of 12-h day/night cycles, ambient temperature was 23 ± 2°C, with humidity of 35 ± 5%. All experiments were carried out in accordance with Chinese national laws and local guidelines. The animal study was approved by the Animal Ethics Committee of Tianjin University of Traditional Chinese Medicine (approval number TCM-2012-078F01).

#### Preparation of Guinea Pig Anti-mouse Platelet Serum (GP-APS)

##### Antigen preparation

Mouse blood obtained by retro-orbital bleeding was mixed with EDTA-Na_2_ anticoagulant, and platelets separated by gradient centrifugation. Samples were then diluted with saline solution and after microscopic observation, their concentration was adjusted to 1 ∼ 2 × 10^9^/L.

##### Immunological methods

Platelet suspensions were diluted 1:1 in incomplete Freund’s adjuvant, or in complete Freund’s adjuvant (water-in-oil emulsion). Complete Freund’s adjuvant antigen was injected subcutaneously into guinea pigs’ foot, palm, and back, (100 μL per injection site) at 0 weeks. Antigens in incomplete Freund’s adjuvant were subcutaneously injected into the same anatomical locations at 1, 2, and 4 weeks. At week 5, blood was drawn from the guinea pigs’ hearts and let to coagulate at room temperature for 1 h. Blood samples were placed at 4°C overnight, centrifuged at 2,000 rpm × 20 min and the supernatant, consisting of GP-APS was placed at -20°C until use (Zhang et al., 2016).

##### Antiserum treatment

GP-APS was removed from the freezer, thawed, and placed in a water bath at 56°C for 30 min. After adsorption with the same amount of BALB/c mouse red blood cells, GP-APS was diluted 1:4 (v:v) with saline (Zhang et al., 2016).

#### *In Vivo* ITP Model and Treatment

Sixty mice were randomly divided into six groups (10 mice/group): (1) control; (2) model control; (3) low-dose ZDF treatment; (4) middle-dose ZDF treatment; (5) high-dose ZDF treatment; and (6) prednisone acetate treatment. Except for the control group, which received saline, the other groups were injected intraperitoneally with 1:4 (v:v) GP-APS at 0, 1, 3, 5, 7, 9, 11, and 13 days to model ITP. ZDF was administered to the low-, middle-, and high-treatment groups. Prednisone acetate was administered to prednisone acetate treatment group. Twenty-four h after the last GP-APS injection, the mice were given intragastric administration once a day for 14 consecutive days, and the animals were sacrificed on the 15th day after sampling (Yang et al., 1994). Details are shown in **Table 1**.

**Table 1:** Animal experiment grouping and dosing regimen.

Grouping	Number	Drug	Dose	Mode of administration	Sampling time (day)
Control group	10	Normal saline	5 mL/kg/day	i.p., successive administration	8
		Distilled water	10 mL/kg/day	i.g., successive administration	14
Model group	10	GP-APS	5 mL/kg/day	i.p., next day administration	8
		Distilled water	10 mL/kg/day	i.g., successive administration	14
ZDF high dose group	10	GP-APS	5 mL/kg/day	i.p., next day administration	8
		ZDF-H	16.8 g/kg/day	i.g., successive administration	14
ZDF middle dose group	10	GP-APS	5 mL/kg/day	i.p., next day administration	8
		ZDF-M	8.4 g/kg/day	i.g., successive administration	14
ZDF low dose group	10	GP-APS	5 mL/kg/day	i.p., next day administration	8
		ZDF-L	4.2 g/kg/day	i.g., successive administration	14
Prednisone acetate group	10	GP-APS	5 mL/kg/day	i.p., next day administration	8
		Prednisone acetate	7.8 mg/kg/day	i.g., successive administration	14

*i.p., intraperitoneal injection; i.g., oral administration by gavage.*

#### Sample Collection

After 7 and 14 days of ITP modeling, peripheral blood platelet numbers were measured using an automatic blood cell analyzer. After 14 days of modeling, blood was taken without anticoagulation by retro-orbital bleeding, and centrifuged at 3,500 rpm for 10 min to separate the serum, which was stored at -80°C for metabolic studies. On day 15, mice were sacrificed and the spleens were dissected, weighed, and quickly transferred to a -80°C freezer, for target protein detection. The right femurs were stripped, their ends cut off, and the bone marrow was repeatedly flushed out into a 1.5 mL sample tube using a small amount of saline. After centrifugation at 2,000 rpm for 10 min, the supernatant was discarded and a smear of the lower layer of bone marrow cells was fixed in methanol, stained in Swiss-Giemsa, and sealed with neutral gum for bone marrow megakaryocyte count.

### Western Blot Analysis

Spleen tissue was homogenized, and equal amounts of protein (quantified by the BCA method) were separated by 12% SDS-PAGE, transferred to a PVDF membrane, and then blocked with 5% BSA. Primary antibodies were incubated overnight, followed by TBST washing and incubation with secondary antibodies for 1 h. After washing, 200 μl of luminol were added for UVP chemiluminescence detection.

### ELISA Analysis

The levels of serum cytokines (IL-2, IFN-γ, TPO, GM-CSF, TNF-α, and IL-6) were detected by ELISA kit according to the manufacturer’s instructions. The kit was removed from the refrigerator at 4°C centigrade and balanced at room temperature for 20 min. Standard wells, sample wells and blank wells were set. Blank hole as a reference. Standard wells were added to different concentration of standard 50 μL. Sample wells were added to the sample diluent 40 μL, after adding the sample to be tested 10 μL. Subsequently, the standard wells and sample wells were added to the HRP labeled detection antibody 100 μL. The wells were sealed with a sealing film and incubated at 37°C for 60 min. Then discard the liquid, added washing liquid and repeated washing five times. After the liquid was dried, all the wells were added to the substrate A and B each 50 μL, and incubated at 37°C for 15 min protect form light. Subsequently, 50 μL of the stop solutions were added to all the wells, and the absorbance (OD) value of each well was measured at a wavelength of 450 nm. According to the OD value and concentration of the standard wells, obtained the standard linear regression equation of each enzyme, and calculated the concentration of each sample hole. Based on the concentration of each sample well, the average value of the contents of each group of related enzymes, the change in content and the significance with respect to the control group were calculated.

### Metabolomics Analysis

#### Sample Preparation

Following thawing at room temperature, 100 μl aliquots of serum samples were mixed with 300 μl of acetonitrile, sonicated for 10 min on ice, centrifuged at 13,000 rpm for 15 min at 4°C, and the supernatants analyzed by UPLC-Q-TOF/MS.

#### QC Sample Preparation

To ensure the stability and repeatability of the systems, plasma samples singled out from each group were pooled as a quality control (QC) sample, mixed for 1 min, and centrifuged at 13,000 rpm for 10 min to obtain supernatants.

### UPLC-Q-TOF/MS Serum Analysis

#### Chromatographic Conditions

ACQUITY UPLC HSS C18 columns (2.1 × 100 mm, 1.8 μm, Waters); flow rate, 0.3 mL/min; column temperature, 40°C; injection volume, 5 μL. For the gradient elution process, the A phase was 0.1% formic acid/water, the B-phase was 0.1% formic acid/acetonitrile. The gradient started with 99% A, followed by 0–0.5 min, A: 99 to 99%; 0.5–2 min, A: 99 to 50%; 2 to 9 min, A: 50 to 1%; 9–10 min, A: 1 to 1%; 10– 10.5 min, A: 1 to 99%; and 10.5–12 min, A: 99 to 99%.

#### MS Conditions

Using ESI as source, mass spectral analyses were carried out in positive ionization mode. High-purity N_2_ was used as auxiliary ESI gas for solvent removal: drying gas flow, 10 ml/min; N_2_ temperature, 325°C; pressure of atomized gas was 350 psi; desolvation gas flow, 600 L/h; capillary voltage, 3.5 kV; data acquisition range was 50–1000 Da.

### Methodological Validation

For instrument precision testing, the same QC sample solutions were continuously injected 6 times, and 20 chromatographic peaks were randomly selected. Relative standard deviation (RSD) values of peak area and retention time of the 20 chromatographic peaks were calculated.

For method precision testing, 6 QC samples were prepared in parallel, and 20 chromatographic peaks were randomly selected for continuous injection analysis. The RSD values of the peak area and retention time of the 20 chromatographic peaks were calculated.

For sample stability testing, the same QC sample solution was used for 0, 6, 12, 18, and 24 h injection analyses, and 20 chromatographic peaks were randomly selected. RSD values of corresponding peak area and retention times were calculated.

### Metabolomics Data Processing

Mice serum were analyzed by UPLC-Q-TOF/MS. Multivariate statistical analysis and data integration analysis were used to screen terminal metabolites differentially regulated by ZDF in ITP mice. The data analysis process was as follows: first, multivariate data analysis was performed using SIMCA-P +11.5 software. PCA was used to remove outlier samples. Second, supervised PLS-DA was performed, and compounds that had a significant contribution to the classification (VIP > 1) were considered as differentially regulated candidate metabolites. Finally, Independent Samples *t*-test was performed using SPSS 17.0, and *p* < 0.05 indicated significance. We identified potential biomarkers based on their m/z values, the HMDB^2^, and the KEGG database and literature^3^. The differential metabolites of ITP mice obtained upon final identification were searched in the drug delivery group, and the effects of ZDF on these metabolites were recorded.

### Statistical Analysis

Data were expressed as mean ± standard deviation (SD) and analyzed with SPSS software. Statistically significant values were carried out by one-way ANOVA test with *post hoc* contrasts by Student–Newman–Keuls test. *P* < 0.05 indicated statistical significance.

## Results

### Identification of Zi Dian Fang’s Chemical Constituents and Attribution Analysis

**FIGURE 1:**
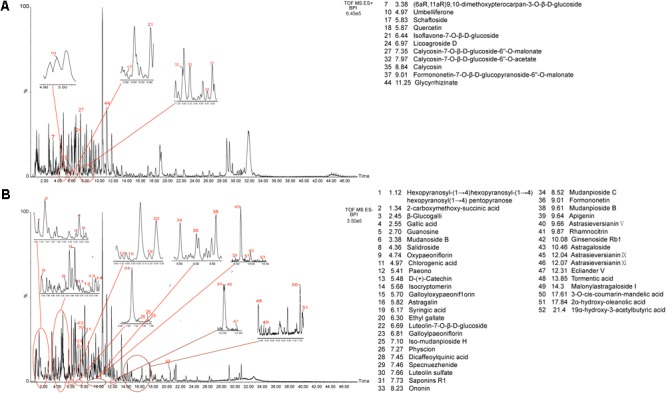
Total ion flow chart of Zi Dian Fang. **(A)** ESI (+); **(B)** ESI (–).

For comprehensive identification of ZDF chemical constituents, a ZDF test solution was analyzed using UPLC-Q-TOF/MS both in positive and negative ion scanning modes. The corresponding total ion current diagrams are shown in **Figures 1A,B**. The total ion current diagram of single herbs is shown in Supplementary Figures S1A,B. A total of 52 ZDF constituents were identified, and later subjected to network pharmacological analysis (Supplementary Table S1).

### Network Pharmacology Analysis

#### Potential Targets

Five hundred and twenty ZDF targets were predicted using the Pharm Mapper server^4^, 45 of which were closely related to ITP with a total frequency of 235 (**Table 2**). Analysis of “chemical composition-target” correlation revealed multiple compounds acting on the same target, and multiple targets affected by the same compound. For instance, the GTPase HRas (Ras) is the target of gallic acid, paeonol, chlorogenic acid, and other chemical components. Astragaloside A simultaneously acts on mitogen-activated protein kinase (MAPK)-activated protein kinase 2, tyrosine-protein kinase ITK/TSK, and protein kinase C-theta, among many other targets. Among the 45 targets closely related to ITP, the Ras target protein appeared with the highest frequency, and it was strongly correlated to platelet proliferation, immunity, and inflammation (Kakunaga et al., 2004; Gunderson et al., 2013; Hauling et al., 2014).

**Table 2:** Zi Dian Fang (ZDF) major active ingredient related targets.

Serial number	Uniport ID	Target protein name	Frequency
1	P01112	GTPase HRas	35
2	Q9BZX2	Uridine-cytidine kinase 2	26
3	P09211	Glutathione S-transferase P	17
4	P27707	Deoxycytidine kinase	14
5	P18031	Tyrosine-protein phosphatase non-receptor type 1	10
6	P29218	Inositol monophosphatase 1	11
7	P24941	Cell division protein kinase 2	9
8	Q08499	cAMP-specific 3,5-cyclic phosphodiesterase 4D	9
9	P49841	Glycogen synthase kinase-3 beta	8
10	P08473	Neprilysin	9
11	P12931	Proto-oncogene tyrosine-protein kinase Src	7
12	Q02750	Dual specificity mitogen-activated protein kinase kinase 1	6
13	P08246	Neutrophil elastase	5
14	Q00534	Cell division protein kinase 6	5
15	O15530	3-phosphoinositide-dependent protein kinase 1	4
16	Q16539	Mitogen-activated protein kinase 14	4
17	P35558	Phosphoenolpyruvate carboxykinase, cytosolic [GTP]	4
18	P15121	Aldose reductase	5
19	Q06187	Tyrosine-protein kinase BTK	3
20	P02679	Fibrinogen gamma chain	3
21	P11712	Cytochrome P450 2C9	3
22	P00492	Hypoxanthine-guanine phosphoribosyltransferase	3
23	P36873	Serine/threonine-protein phosphatase PP1-gamma catalytic subunit	3
24	P49137	MAP kinase-activated protein kinase 2	3
25	Q08881	Tyrosine-protein kinase ITK/TSK	3
26	P20248	Cyclin-A2	2
27	P11362	Basic fibroblast growth factor receptor 1	2
28	Q03518	Antigen peptide transporter 1	2
29	P00918	Carbonic anhydrase 2	2
30	P53779	Mitogen-activated protein kinase 10	2
31	P11309	Serine/threonine-protein kinase pim-1	2
32	Q04759	Protein kinase C theta type	1
33	P14555	Phospholipase A2, membrane associated	1
34	P08709	Coagulation factor VII	1
35	P03951	Coagulation factor XI	1
36	P35968	Vascular endothelial growth factor receptor 2	1
37	P31749	RAC-alpha serine/threonine-protein kinase	1
38	P42574	Caspase-3	1
39	O60760	Glutathione-requiring prostaglandin D synthase	1
40	P42330	Aldo-keto reductase family 1 member C3	1
41	P15056	Serine/threonine-protein kinase B-raf	1
42	Q13231	Chitotriosidase-1	1
43	P28161	Glutathione S-transferase Mu 2	1
44	Q00688	Peptidyl-prolyl *cis*-trans isomerase FKBP3	1
45	P08581	Hepatocyte growth factor receptor	1

### Zi Dian Fang Correlative Pathways and Construction of “Chemical Components-Targets-Pathways” Regulatory Network

**FIGURE 2:**
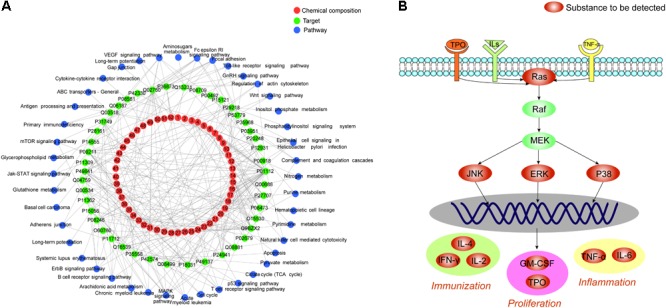
**(A)** Zi Dian Fang’s “chemical composition-target-pathway” regulatory network. Numbers 1–52 circled in red represent chemical composition, and correspond to data on **Figure 1**. **(B)** Identified Ras/MAPKs signaling pathway proteins.

Based on the above results, cellular pathways were analyzed by MAS 3.0^5^ and the KEGG database^6^, and 113 pathways were identified. Among these, 43 were closely related to ITP. Pathways associated with platelet proliferation included ‘hematopoietic cell lineage,’ ‘complement and coagulation cascade,’ and ‘JAK-STAT signaling pathway,’ among others (Morin-Poulard et al., 2013; Conway, 2015; Guo et al., 2015). Pathways associated with immunoregulation included ‘B cell receptor signaling pathway,’ ‘T cell receptor signaling pathway,’ ‘antigen processing and presentation,’ ‘natural killer cell-mediated cytotoxicity,’ and ‘Toll-like receptor signaling pathway,’ among others (Notarangelo, 2014; Zhong et al., 2014; Roche and Furuta, 2015; Goulopoulou et al., 2016; Rajalingam, 2016). Inflammation-related pathways included ‘MAPK signaling pathway,’ ‘VEGF signaling pathway,’ and ‘AA metabolism’ (Ramakrishnan et al., 2014; Schuck et al., 2014; Peng et al., 2016). Cytoscape software was used to establish the “chemical composition-target-pathway” regulatory network of ZDF, which shows the correlation of 52 compounds, 45 target proteins, and 43 ITP-associated pathways (**Figure 2A**).

There were 27 pathways associated with Ras protein, among which ‘B cell receptor signaling pathway,’ ‘T cell receptor signaling pathway,’ and ‘MAPK signaling pathway’ were the most closely related ones. KEGG data visualization made it obvious that the Ras downstream target proteins p38, ERK, and JNK are key connection points between the MAPK signaling pathway and both T cell and B cell receptor signaling pathways. Activation of p38, ERK and JNK can affect cell proliferation, immune status, and inflammation. According to the results of network pharmacology, a preliminary prediction of the mechanism by which ZDF treats ITP may be as follows: ZDF inhibits the expression of Ras and prevents phosphorylation of its downstream effectors ERK, JNK, and p38, thus promoting platelet proliferation, immune regulation, and an anti-inflammatory effect. Subsequently, for network pharmacology validation an animal model of ITP was established to detect the key targets in the Ras/MAPKs signaling pathway (**Figure 2B**), and to observe variation trends of the detection indexes.

### *In Vivo* Verification of Network Pharmacology-Based Virtual Screening Results

#### Effect of Zi Dian Fang on Peripheral Platelet Count, Spleen Index, and Bone Marrow Megakaryocytes Count

The effect of ZDF on peripheral platelet counts was evaluated in ITP mice on days 7 and 14 after ITP induction by GP-APS injection. Platelet counts in the model group were significantly lower than in the control group. Meanwhile, ITP mice treated with ZDF exhibited increased peripheral platelet counts compared with the model group (Supplementary Table S2). The splenic index in the ITP model group was significantly higher than in the control group, and was reduced by ZDF treatment (Supplementary Table S3), indicating effective reduction of ITP-induced splenomegaly in ITP mice. The total number of bone marrow megakaryocytes was increased in the ITP model group compared with control mice. Specifically, the number of thrombogenic megakaryocytes was decreased and the number of granular megakaryocytes was increased. The total number and classification of bone marrow megakaryocytes in the different groups are listed in Supplementary Table S4. These results suggest active bone marrow megakaryocyte proliferation in ITP mice, accompanied by anomalous maturation reflected by increased numbers of granular megakaryocytes and decreased numbers of thrombogenic megakaryocytes. ZDF administration largely normalized these changes, indicating a therapeutic effect.

While prednisone’s beneficial effects on platelet and megakaryocyte counts were similar to those of ZDF, analysis of the spleen index showed that prednisone acetate significantly decreased spleen index compared with the control group, but the spleen index in the treatment group did not decrease significantly compared with the control group. Thus, ZDF may have the advantage of alleviating the adverse effects of immunosuppression associated with prednisone use.

### Western Blot and Enzyme-Linked Immunosorbent Assay Analysis

Ras signaling pathway plays a critical role in the T and B cell receptor and MAPK signaling pathways. The Ras/MAPK pathway effectors ERK1/ERK2, JNK, and p38 are important members of the MAPK family (Gao et al., 2017). Under normal physiological conditions, these proteins are dephosphorylated and signal transduction does not occur. When cells are stimulated (i.e., during inflammation, immune challenge, etc.), Ras activation leads to downstream ERK, JNK, and p38 phosphorylation and activation of the Ras/MAPKs signaling pathway, which promotes the expression of cell factors associated with immunoregulation (IL-2, IFN-γ), platelet proliferation (TPO, GM-CSF), and inflammation (TNF-α, IL-6) (Kitanaka et al., 2017).

**FIGURE 3:**
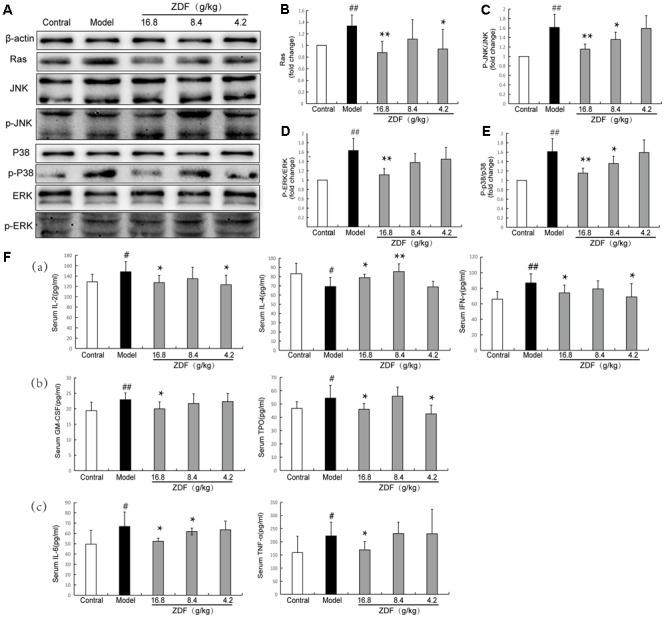
Western blot and ELISA studies. **(A)** Representative western blots of Ras, JNK, p-JNK, p38, p-p38, ERK, and p-ERK proteins. **(B–E)** Relative Ras protein expression level and p-JNK/JNK, p-p38/p38, p-ERK/ERK ratios in the experimental animal groups. **(F)** ELISA evaluation of serum concentrations of IL-2, IL-4, IFN-γ, GM-CSF, TPO, TNF-α, and IL-6 in experimental animal groups. (a) Immunity-related indexes: IL-2, IL-4, and IFN-γ. (b) Proliferation-related indexes: TPO and GM-CSF. (c) Inflammation-related indexes: TNF-α and IL-6. ^#^*P* < 0.05, ^##^*P* < 0.01, compared with the control group; ^∗^*P* < 0.05, ^∗∗^*P* < 0.01, compared with the model group.

To correlate ZDF network pharmacology predictions with *in vivo* ZDF effects, splenic levels of Ras, ERK, JNK, and p38, and their corresponding phosphorylated forms were verified by western blot. Compared with the control group, Ras, p-JNK/JNK, p-ERK/ERK, and p-p38/p38 levels were significantly increased in the model group, while these protein expression changes were attenuated by treatment with ZDF (**Figures 3A–E**). Next, ELISA assays were carried out in serum samples to analyze molecular markers associated with proliferation, i.e., TPO and GM-CSF, inflammation, i.e., TNF-α and IL-6, and immune response, i.e., IL-2, IFN-γ and IL-4. As shown in **Figure 3B**, IL-2, IFN-γ, TPO, GM-CSF, TNF-α, and IL-6 levels were significantly increased, while IL-4 production was significantly decreased, in the model group compared with the control group, while all these substances were differentially regulated in the ZDF treatment group.

### Metabolomics Analysis

Method precision and sample stability tests were first carried out using serum QC samples in positive ion mode. The base peak chromatogram (BPC) is shown in Supplementary Figure S2. Results showed that peak area were less than 15% and retention time RSD values were less than 5%, indicating that instrument precision, method precision and sample stability were reliable. Specific information is shown in Supplementary Table S5.

**FIGURE 4:**
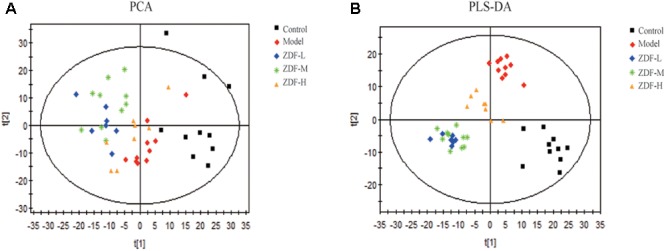
Multivariate analysis results. **(A)** PCA score plots of the model group compared with the control group, and the ZDF-L, ZDF-M, and ZDF-H treatment groups compared with the model group. **(B)** PLS-DA score plots of the model group compared with the control group, and the ZDF-L, ZDF-M, and ZDF-H treatment groups compared with the model group. ZDF-L: ZDF low dose group; ZDF-M: ZDF middle dose group; ZDF-H: ZDF high dose group.

Metabolomics studies were performed using multivariate statistical analysis. First, PCA was used for unsupervised data analysis (**Figure 4A**) but showed no clear distinction between the control, model, and treatment groups. Therefore, we carried out supervised data analysis using PLS-DA (**Figure 4B**), which showed good distinction between the three groups. This analysis indicated that endogenous metabolite levels in the model group were significantly different compared with the control group. After ZDF administration, metabolite levels differed in turn significantly from the model group, approaching the levels observed in the control group. Based on the PLS-DA analysis, variables with VIP > 1 between the control and model groups were selected as potential biomarkers associated with ITP, and their significance assessed through Student’s *t*-test.

Twelve differentially regulated metabolites were identified with basis on their variation in the ZDF treatment groups and available literature reports. Among these, leucine, arginine, palmitoleic acid, stearic acid, and 5-aminoimidazole ribonucleotides, and phenylpyruvic acid levels were significantly decreased, while S1P, 9-*cis*-retinoic acid, EPA, and Δ^12^-PGJ2 levels were significantly increased in the model group compared with the control group. All these metabolites were differentially regulated in the ZDF treatment group. Detailed information is shown in **Table 3**.

**Table 3:** Identified potential biomarkers for the discrimination between model group and control group in serum samples.

No.	Rt (min)	(m/z)	Adduct	Metabolites	Formula	Trend		
						Model Group vs. control group	ZDF-L vs. Model Group	ZDF-M vs. Model Group	ZDF-Hvs. Model Group
1	1.20	296.065	M+H	5-aminoimidazole ribonucleotide	C_8_H_14_N_3_O_7_P	↓^##^	↓	↓	↑^∗^
2	1.24	198.0859	M+Na	Argininic acid	C_6_H_13_N_3_O_3_	↓^##^	↑	↑	↑
3	1.56	165.0573	M+H	Phenylpyruvic acid	C_9_H_8_O_3_	↓^##^	↑	↑^∗∗^	↑^∗^
4	1.56	132.101	M+H	(±)-leucine	C_6_H_13_NO_2_	↓^##^	↑^∗^	↑^∗∗^	↑^∗∗^
5	4.54	357.2039	M+Na	Delta-12-prostaglandin j2	C_20_H_30_O_4_	↑^#^	↑	↑	↓^∗^
6	5.44	402.2389	M+Na	Sphingosine 1-phosphate	C_18_H_38_NO_5_P	↑^#^	↓	↓	↓^∗^
7	6.05	301.2173	M+H	9-cis-Retinoic acid	C_20_H_28_O_2_	↑^##^	↑^∗^	↑	↑
8	6.39	263.2389	M+Na	Palmitaldehyde	C_16_H_32_O	↓^##^	↑	↑^∗^	↑^∗^
9	6.88	282.2792	M+H	Oleamide	C_18_H_35_NO	↑^#^	↓^∗^	↓	↓
10	8.68	277.2144	M+Na	Palmitoleic acid	C_16_H_30_O_2_	↓^##^	↑	↑^∗^	↑^∗∗^
11	8.68	277.2144	M+H	Stearidonic acid	C_18_H_28_O_2_	↓^##^	↑^∗∗^	↑^∗^	↑^∗∗^
12	9.18	303.2316	M+H	Eicosapentaenoic acid	C_20_H_30_O_2_	↓^#^	↑	↓	↑^∗^

*^#^Represents the changes in the model groupcompared with control group, *P* < 0.05; ^##^represents the changes in the model groupcompared with control group, *P* < 0.01; ^∗^represents the changes in the treatment groupcompared with model group, *P* < 0.05; ^∗∗^represents the changes in the treatment groupcompared with model group, *P* < 0.01; ↑, content increased; ↓, content decreased. “vs.” means “versus”; “ZDF-L” means “ZDF low dose group”; “ZDF-M” means “ZDF middle dose group”; “ZDF-H” means “ZDF high dose group”.*

### Network Pharmacology and Metabolomics Integration Analysis

**FIGURE 5:**
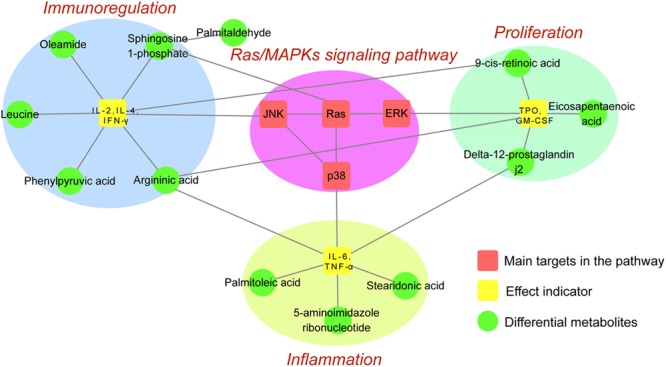
Network map of the Ras/MAPKs signaling pathway and differential metabolites.

To integrate network pharmacology and metabolomics data, main target proteins and differentially regulated metabolites within the Ras/MAPKs signaling pathway were respectively screened by these methods. Then, the ID number of target proteins and differential metabolites were searched on the KEGG database to find shared metabolic pathways, and results combined with the literature to find reported relationships. Overall analysis results preliminarily confirmed that ZDF therapeutic actions on ITP are based on inhibition of the Ras/MAPKs signaling pathway and attenuation of immune and inflammatory responses and promotion of platelets proliferation. The correlation between target proteins and differential metabolites affected by ZDF is illustrated in **Figure 5**.

## Discussion

### Platelet Homeostasis and Proliferation

Through different techniques, we demonstrated that the level of Ras and p-ERK/ERK in spleen of the model group was significantly higher than that in the control group (**Figures 3B,D**), serum TPO, GM-CSF, S1P, EPA, and Δ^12^-PGJ2 levels were significantly increased and arginine was significantly decreased in the model group compared with the control group, while all these substances were differentially regulated in the ZDF treatment group.

Ras protein is a key upstream protein in the Ras/MAPKs signaling pathway and it is an important component in regulating cell growth, proliferation and differentiation. ERK is downstream protein of the Ras/MAPKs signaling pathway. S1P is a biologically active sphingolipid that acts as an extracellular ligand for S1P membrane receptors (S1P1-5) and affects cell survival, proliferation, and differentiation through distinct signal transduction pathways (Mendelson et al., 2014). TPO is a specific growth factor that stimulates proliferation and differentiation of megakaryocytes and hence regulates platelet production (Mazza et al., 2016). When the number of peripheral platelets is high, TPO uptake increases, serum TPO decreases, and proliferation, differentiation, and maturation of megakaryocytes is inhibited, resulting in decreased platelet production (Hitchcock and Kaushansky, 2014). In the course of ITP, peripheral platelet levels decrease and serum TPO increases. TPO binds to its receptor and activates the Ras/ERK signaling pathway, leading to S1P generation (Garcia et al., 2001). S1P binds to its G protein-coupled receptor(s) and further activates the Ras/ERK signaling pathway, leading to an increase in GM-CSF, which in turn stimulates differentiation of myeloid progenitor cells (Na et al., 2016; Kraakman et al., 2017). Elevated levels of TPO and GM-CSF also promote macrophage maturation. Activated macrophages are classified into two types: classically activated macrophages (M1) and alternatively activated macrophages (M2) (Genin et al., 2015). M1 cells contain NOS, which catalyzes the oxidation of the essential amino acid arginine to NO, which inhibits platelet aggregation (Mazzanti et al., 2011; Roy et al., 2015). The same effect is elicited by EPA, one of the most abundant and essential unsaturated fatty acids (Ohnishi and Saito, 2013). EPA-mediated inhibition of platelet aggregation is related to the metabolism of AA (Roy et al., 2016). AA is metabolized by COX to form two substances with opposing actions: PGI2, a Δ^12^-PGJ2 precursor that inhibits platelet aggregation, and TXA2, which is vasoconstrictor and promotes platelet aggregation (Kashiwagi et al., 2015). Studies have shown that EPA can inhibit the formation of TXA2, thereby inhibiting platelet aggregation (Schmitz et al., 2000). However, while NO and EPA both inhibit platelet aggregation, a simultaneous rise in these two substances can lead to coagulation dysfunction and aggravate symptoms in ITP patients.

Our data suggests that the mechanism by which ZDF promotes platelet proliferation and improves coagulation in patients with ITP may be as follows: on the one hand, ZDF reduces serum TPO concentration and inhibits Ras/ERK signaling pathway activation, so that downstream GM-CSF and S1P expression decreases, resulting in reduced platelet activation and increased platelet proliferation. On the other hand, ZDF-mediated Ras/ERK signaling inhibition reduces the number of M1 cells, leading to lower NOS activity, reduced arginine consumption and NO production, and reduced EPA and Δ^12^-PGJ2 synthesis through AA metabolism. All these changes counteract defective platelet aggregation and improve coagulation, thus reducing ITP symptoms.

### Immunoregulation

Present results showed that the concentration of immune-related cytokines IL-2 and IFN-γ in the serum of ITP mice was significantly higher than in the control group, while the content of IL-4 was instead decreased. In addition, leucine, arginine, and phenylpyruvic acid levels were decreased (**Figure 3F**), while S1P, 9-*cis*-retinoic acid, and oleic acid amide levels were increased, in the model group. Levels of all these substances were differentially regulated in the ZDF treatment group.

Immune thrombocytopenic purpura etiology is closely related to abnormal immune function, particularly involving Th1/Th2 cell dysfunction (Yao et al., 2014). IL-2, IFN-γ, and IL-4 synthesis is regulated by the Ras/MAPKs signaling pathway in Th1 and Th2 cells. In general terms, IL-2 and IFN-γ are produced by activated Th1 cells and mediate cellular immune responses, while IL-4 is produced by activated Th2 cells and mediates humoral immune responses (Hall, 2015; Almeida et al., 2017). Leucine is the most common amino acid found in proteins, and can indirectly promote the release of IL-4 (Sahoo et al., 2015). Consistent with Th1/Th2 dysfunction, leucine deficiency leads to decreased IL-4 production, weakening of the humoral immune response, and stimulation of the cellular immune response (Bassit et al., 2002). S1P is widely found in blood, lymph, erythrocytes, neutrophils, platelets, and other cells. S1P4 receptor balances Th1/Th2 functions by inhibiting IL-4 secretion by Th2 cells and stimulating IL-10 production by Th1 cells (Wang et al., 2005; Walsh et al., 2014). Differential metabolism of arginine can also enhance the activity of Th cells by stimulating NOS-mediated NO production, which promotes apoptosis and inhibits proliferation and activation of T cells (Bonham et al., 2010; Raber et al., 2014). Reduced T cell proliferation and activation can also be elicited by oleamide, a long-chain fatty acid amide that increases intracellular Ca^2+^ concentration in T cells (Murillo-Rodríguez et al., 2001). KEGG pathway database analysis indicated that increases in IL-2 and IFN-γ are associated with increased 9-*cis*-retinoic acid content, leading to an imbalance in Th1/Th2 activities. Meanwhile, the metabolism of phenyl pyruvate, another metabolite found to be decreased in our ITP model, is closely related to that of tryptophan, which has been linked to immune function regulation (Capuron et al., 2014).

Our analyses suggest that ZDF balances Th1/Th2 activities in patients with ITP by inhibiting the release of the Th1-type cytokines IL-2 and IFN-γ (thus reducing 9-*cis*-retinoic acid levels), by promoting IL-4 release, and by inhibiting IL-10 release by upregulation of arginine and downregulation of S1P and oleic acid amide. On the other hand, ZDF may improve the immune response by upregulating phenylpyruvic acid synthesis.

### Anti-inflammatory Effects

Ras/MAPKs signaling activation is closely linked to inflammatory stimuli (Kim et al., 2015; Hou et al., 2016), leading to production of cytokines such as IL-6 and TNF-α (Li et al., 2016). IL-6 is a lymphokine produced by activated T cells and fibroblasts, which regulates the inflammatory response (Chaturvedi et al., 2015). Starkie et al. indicate that skeletal muscle markedly releases IL-6, which could exert anti-inflammatory effects through the downregulation of TNF-α activity during “non-damaging” exercise (Starkie et al., 2003). When ITP occurs, the body is in an inflammatory state (Dal et al., 2015). In the course of inflammation, naive T cells differentiate into Th17 cells in the presence of TGF-β1 and IL-6. IL-6 inhibits the development of Treg cells and promotes the development of inflammatory responses (Bettelli et al., 2006). In our study, serum IL-6 and TNF-α levels were significantly increased in ITP mice (**Figure 3F**), which is consistent with the literature reports (Talaat et al., 2014). We consider that IL-6 may play a pro-inflammatory role in the ITP model. TNF-α has critical functions in the inflammatory microenvironment, and its cytotoxic effect is related to activation of phospholipase A2, which releases AA from membrane phospholipids (Ahn et al., 2015). AA, which may also be generated from stearic acid desaturation to oleic acid (Harvey et al., 2010), can lead to oxidative stress and initiation of chronic inflammatory responses by its metabolism through COX and lipoxygenase (LOX). Since COX-2 is the rate-limiting enzyme in the synthesis of prostaglandins from AA in macrophages, leukocytes, and fibroblasts, we speculate that the observed downregulation of stearic acid and upregulation of Δ^12^-PGJ2 expression in ITP mice may be related to COX-2 overexpression. Δ^12^-PGJ2 is a potent inflammatory mediator that promotes the release of IL-6 and TNF-α (Heinemann et al., 2003). Inflammation also stimulates iNOS in immune and endothelial cells, which generate large amounts of NO that positively affect further expression of iNOS and exacerbate inflammation (Guzik et al., 2003). Palmitoleic acid (9-hexadecenoic acid) belongs to the same unsaturated fatty acid family of stearic acid. Both fatty acids were down regulated in model group mice, suggesting that impaired lipid metabolism may contribute to the inflammatory response in ITP mice (van Diepen et al., 2013).

Based on the present findings, we suggest that ZDF reduces inflammation in ITP mice by lowering the production of pro-inflammatory cytokines TNF-α and IL-6 through inhibition of p38, ERK, and JNK activation, downregulation of Δ^12^-PGJ2, and upregulation of palmitoleic acid, stearic acid, and arginine levels.

## Conclusion

**FIGURE 6:**
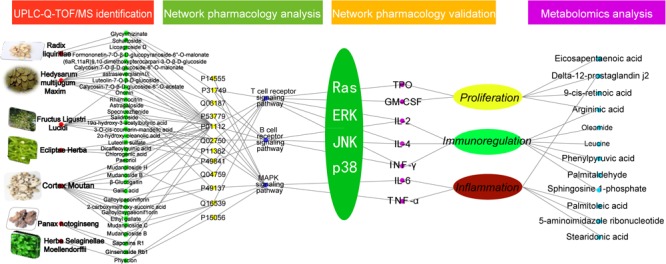
Network pharmacology prediction and metabolomics analysis.

In conclusion, using UPLC, mass spectroscopy-based metabolomics, western blot, ELISA, and network pharmacology the present study analyzed the composition of the TCM prescription ZDF, and the changes it induced on serum metabolites and cellular signaling pathways in an animal model of ITP. Following identification of 52 chemical constituents in the ZDF formulation by UPLC-Q-TOF/MS, network pharmacology predicted significant pharmacological actions centered on three main pathophysiological aspects: platelet proliferation, immunoregulation, and inflammation. After validation of predicted main targets and pathways through animal experiments, we proposed a plausible mechanism for the multi-target effects of ZDF on ITP (**Figure 6**). According to the proposed model, ZDF inhibits Ras expression and phosphorylation of its downstream effectors, ERK, JNK, and p38, attenuating the release of cell factors associated with proliferation (TPO, GM-CSF), inflammation (TNF-α, IL-6), and cell-mediated immunity (IL-2, IFN-γ). Meanwhile, stimulation of IL-4 production promotes platelet proliferation, balances Th1/Th2 immune responses, and reduces inflammatory reactions. This study provides data support for the in-depth study of the mechanisms of ZDF in the treatment of ITP and lays a foundation for the clinical application of ZDF. We believe that ZDF provides an effective strategy and may be a useful alternative to hormone-based therapies for the treatment of ITP.

## Author Contributions

YuL and YWa helped with study design, data interpretation and writing of manuscripts. WL and HL help statistical analysis and editing manuscripts. YaL and HL help to experiment, edit manuscripts, and interpret data. YWu, WD, and GB help writing manuscripts. YZ designed, performed and funded all experiments, analyzed and interpreted data and wrote the manuscript.

## Conflict of Interest Statement

WL and HL was employed by Tasly Pharmaceutical Group. The remaining authors declare that the research was conducted in the absence of any commercial or financial relationships that could be construed as a potential conflict of interest. The reviewer CF and handling Editor declared their shared affiliation.

## Footnotes

1 http://www.uniprot.org/

2 http://www.hmdb.ca/

3 http://www.genome.jp/kegg/

4 http://lilab.ecust.edu.cn/pharmmapper/

5 http://bioinfo.capitalbio.com/mas3

6 http://www.genome.jp/kegg/pathway.html

## Abbreviations

Δ^12^-PGJ2: Δ^12^-prostaglandin J2
AA: arachidonic acid
ANOVA: analysis of variance
COX: cyclooxygenase
ELISA: enzyme-linked immunosorbent assay
EPA: eicosapentaenoic acid
ERK: extracellular-signal regulated protein kinase
ESI: electrospray ionization
GM-CSF: Granulocyte-macrophage colony stimulating factor
GP-APS: guinea pig anti-mouse platelet serum
HMDB: Human Metabolome Database
HPA: hypothalamic-pituitary-adrenal
HRP: horseradish peroxidase
IFN-γ: interferon gamma
IL-2: interleukin-2
IL-4: interleukin-4
IL-6: interleukin-6
ITP: immune thrombocytopenic purpura
iNOS: inducible nitric oxide synthase
JNK: c-Jun N-terminal kinase
KEGG: Kyoto Encyclopedia of Genes and Genomes
LOX: lipoxyoxygenase
MAPK: mitogen-activated protein kinase
NO: nitric oxide
NOS: nitric oxide synthase
p38MAPK: p38 mitogen-activated protein kinase
PCA: principal component analysis
PGI2: prostacyclin
PLS-DA: partial least squares-discriminant analysis
PVDF: Polyvinylidene fluoride
QC: quality control
SDS-PAGE: Sodium dodecyl sulfate-polyacrylamide gel electrophoresis
S1P: Sphingosine 1-phosphate
TCM: traditional chinese medicine
Th1: T helper 1
Th2: T helper 2
TNF-α: tumor necrosis factor alpha
TPO: Thrombopoietin
TXA2: thromboxane A2
VIP: Variable Importance Plot
ZDF: Zi Dian Fang
