# Supplementary material for: Integrated Network Pharmacology and Metabolomics Analysis of the Therapeutic Effects of Zi Dian Fang on Immune Thrombocytopenic Purpura
Source: Front Pharmacol. 2018 Jun 19;9:597. doi: 10.3389/fphar.2018.00597 (PMC6018083; doi:10.3389/fphar.2018.00597)
Supplement: Supplementary file 1 [file Presentation_1.pdf]

# Supplementary Material

## Integrated network pharmacology and metabolomics analysis of the therapeutic effects of Zi Dian Fang on immune thrombocytopenic purpura

**Yubo Li[1#], Yamei Li[1#], Wenliang Lu[2], Hongbin Li[2], Yuming Wang[1], Houmin Luo[1], Yuanyuan Wu[1], Wenying Dong[1], Gang Bai[3], Yanjun Zhang[1]***

[1]Tianjin State Key Laboratory of Modern Chinese Medicine, School of Traditional Chinese Materia Medica, Tianjin University of Traditional Chinese Medicine, Tianjin, China

[2]Tasly Institute, Tasly Pharmaceutical Group, Tianjin, China

[3]College of Pharmacy, Nankai University, Tianjin, China

[#] These two authors contributed equally to this work and should be co-first authors.

***Author for correspondence:**

Yanjun Zhang, Tianjin State Key Laboratory of Modern Chinese Medicine, Tianjin University of Traditional Chinese Medicine, 312 Anshan West Road, Tianjin 300193, China. Email: tianjin_tcm001@sina.com

## Results and discussion

## Identification of Zi Dian Fang's chemical constituents and attribution analysis

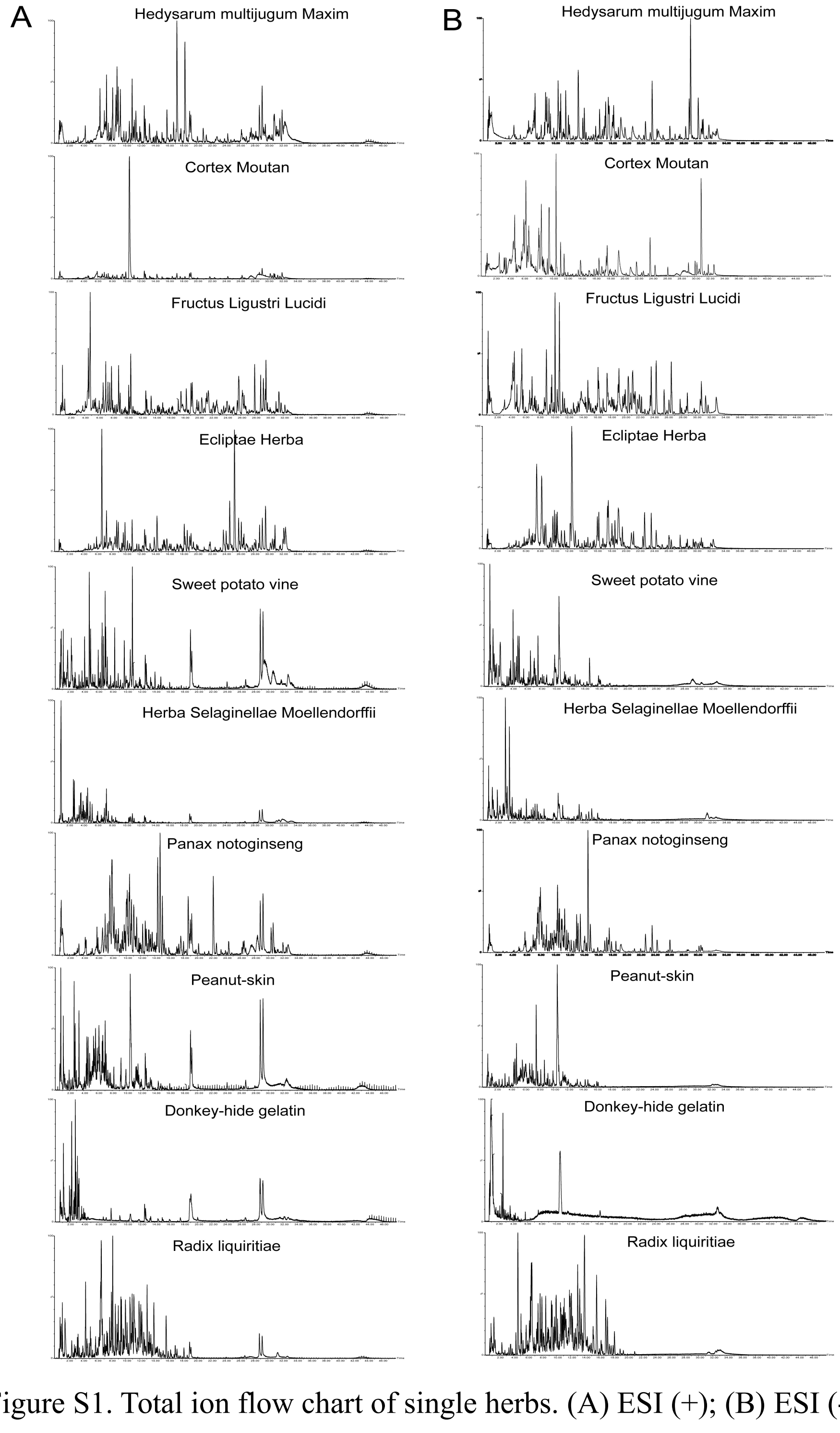


Figure S1. Total ion flow chart of single herbs. (A) ESI (+); (B) ESI (-).

Table S1 Qualitative analysis of the main chemical constituents in ZDF

| Compounds | Retention time(min) | Ionic model | [M+H]/Z [M-H]/Z | Fragment ion(m/z) | Identification | Molecular formula | Source |
|---|---|---|---|---|---|---|---|
| 1 | 1.12 | $[M-H]^-$ | 635.2032 | 455$[M-H-C_6H_{12}O_6]^-$，341$[M-H-C_{11}H_{18}O_9]^-$，353$[M-H-C_{10}H_{18}O_9]^-$，293$[M-H-C_{12}H_{22}O_{11}]^-$，179$[M-H-C_{17}H_{28}O_{14}]^-$， | Hexopyranosyl-(1→4)hexopyranosyl-(1→4)hexopyranosyl(1→4) pentopyranose | $C_{23}H_{40}O_{20}$ | Cortex Moutan |
| 2 | 1.34 | $[M-H]^-$ | 191.0196 | 173$[M-H-H_20]^-$，129$[M-H-CH_2O_3]^-$，111$[M-H-CH_4O_4]^-$ | 2-carboxymethoxy-succinic acid | $C_6H_8O_7$ | Cortex Moutan |
| 3 | 2.45 | $[M-H]^-$ | 331.0645 | 271$[M-H-C_2H_4O_2]^-$，211$[M-H-C_4H_8O_4]^-$，169$[M-H-C_6H_{10}O_5]^-$，125$[M-H-C_7H_{10}O_7]^-$ | β-Glucogalli | $C_{13}H_{16}O_{10}$ | Cortex Moutan |
| 4 | 2.55 | $[M-H]^-$ | 169.0129 | 125$[M-H-CO_2]^-$，107$[M-H-CH_2O_3]^-$ | Gallic acid | $C_7H_6O_5$ | Cortex Moutan |
| 5 | 2.70 | $[M-H]^-$ | 282.0848 | 282$[M-H]^-$，150$[M-H-C_5H_8O_4]^-$ | Guanosine | $C_{10}H_{13}N_5O_5$ | Herba Selaginellae Moellendorffii |
| 6 | 3.38 | $[M-H]^-$ | 463.1083 | 343$[M-H-C_4H_8O_4]^-$，301$[M-H-C_6H_{10}O_5]^-$，241$[M-H-C_8H_{14}O_7]^-$，169$[M-H-C_{11}H_{18}O_9]^-$，125$[M-H-C_{12}H_{18}O_{11}]^-$ | Mudanoside B | $C_{18}H_{24}O_{14}$ | Cortex Moutan |
| 7 | 3.38 | $[M+H]^+$ | 463.1582 | 463$[M+H]^+$，301$[M+H-C_6H_{10}O_5]^+$ | (6aR,11aR)9,10-dimethoxypterocarpan-3-O-β-D-glucoside | $C_{23}H_{26}O_{10}$ | Hedysarum multijugum Maxim |
| 8 | 4.36 | $[M-H]^-$ | 299.1141 | 299$[M-H]^-$， 137$[M-H-C_6H_{10}O_5]^-$ | Salidroside | $C_{14}H_{20}O_7$ | Fructus Ligustri Lucidi |
| 9 | 4.74 | $[M-H]^-$ | 495.1514 | 465$[M-H-CH_2O]^-$，333$[M-H-C_6H_{10}O_5]^-$，281$[M-H-C_{10}H_{14}O_5]^-$，165$[M-H-C_{14}H_{18}O_9]^-$，137$[M-H-C_{16}H_{22}O_9]^-$ | Oxypaeoniflorin | $C_{23}H_{28}O_{12}$ | Cortex Moutan |
| 10 | 4.97 | $[M+H]^+$ | 163.04 | 163$[M+H]^+$，135$[M+H-CO]^+$，107$[M+H-CO-CO]^+$， | Umbelliferone | $C_9H_6O_3$ | Radix liquiritiae |
| 11 | 4.97 | $[M-H]^-$ | 353.0872 | 191$[M-H-C_9H_6O_3]^-$，179$[M-H-C_7H_{10}O_5]^-$ | Chlorogenic acid | $C_{16}H_{18}O_9$ | Sweet potato vine, Ecliptae Herba |

| | | | | | | | |
|---|---|---|---|---|---|---|---|
| 12 | 5.41 | $[M-H]^-$ | 165.0547 | $150[M-H-CH_3]^-$，$135[M-H-C_2H_6]^-$，$122[M-H-C_2H_3O]^-$ | Paeonol | $C_9H_{10}O_3$ | Cortex Moutan |
| 13 | 5.48 | $[M-H]^-$ | 289.0703 | $245[M-H-CHO_2]^-$，$151[M-H-C_7H_6O_3]^-$，$137[M-H-C_8H_8O_3]^-$，$109[M-H-C_9H_8O_4]^-$ | D-(+)-Catechin | $C_{15}H_{14}O_6$ | peanut skin |
| 14 | 5.68 | $[M-H]^-$ | 551.0978 | $267[M-H-C_{16}H_{12}O_5]^-$ | Isocryptomerin | $C_{31}H_{20}O_{10}$ | Herba Selaginellae Moellendorffii |
| 15 | 5.7 | $[M-H]^-$ | 647.1563 | $647[M-H]^-$，$327[M-H-C_7H_5O_5-OCH_2-C_6H_4COOH]^-$ | Galloyloxypaeonif1orin | $C_{30}H_{32}O_{16}$ | Cortex Moutan |
| 16 | 5.82 | $[M-H]^-$ | 447.0923 | $447[M-H]^-$，$285[M-H-C_6H_{10}O_5]^-$，$151[M-H-C_6H_{10}O_5-C_8H_6O_2]^-$ | Astragalin | $C_{21}H_{20}O_{11}$ | Sweet potato vine |
| 17 | 5.83 | $[M+H]^+$ | 565.1572 | $403[M+H-C_6H_{10}O_5]^+$，$271[M+H-C_6H_{10}O_5-C_5H_8O_4]^+$ | Schaftoside | $C_{26}H_{28}O_{14}$ | Radix liquiritiae |
| 18 | 5.87 | $[M+H]^+$ | 303.0493 | $285[M+H-H_2O]^+$，$275[M+H-CO]^+$，$247[M+H-CO-CO]^+$ | Quercetin | $C_{15}H_{10}O_7$ | Sweet potato vine |
| 19 | 6.17 | $[M-H]^-$ | 197.046 | $197[M-H]^-$，$182[M-H-CH_3]^-$ $179[M-H-H_2O]^-$，$164[M-H-CH_3-H_2O]^-$ | Syringic acid | $C_9H_{10}O_5$ | Herba Selaginellae Moellendorffii |
| 20 | 6.3 | $[M-H]^-$ | 197.0458 | $169[M-H-C_2H_4]^-$，$125[M-H-C_3H_4O_2]^-$ | Ethyl gallate | $C_9H_{10}O_5$ | Cortex Moutan |
| 21 | 6.44 | $[M+H]^+$ | 447.1292 | $447[M+H]^+$，$285[M+H-C_6H_{10}O_5]^+$ | Isoflavone-7-O-β-D-glucoside | $C_{22}H_{22}O_{10}$ | Hedysarum multijugum Maxim |
| 22 | 6.69 | $[M-H]^-$ | 447.0918 | $447[M-H]^-$，$285[M-H-C_6H_{11}O_5]^-$ | Luteolin-7-O-β-D-glucoside | $C_{21}H_{20}O_{11}$ | Fructus Ligustri Lucidi |
| 23 | 6.81 | $[M-H]^-$ | 631.166 | $613[M-H-H_2O]^-$，$491[M-H-C_7H_8O_3]^-$，$399[M-H-C_{13}H_{12}O_4]^-$，$313[M-H-C_{17}H_{18}O_6]^-$， | Galloylpaeoniflorin | $C_{30}H_{32}O_{15}$ | Cortex Moutan |
| 24 | 6.97 | $[M+H]^+$ | 449.1466 | $287[M+H-C_6H_{10}O_5]^+$ | Licoagroside D | $C_{22}H_{24}O_{10}$ | Hedysarum multijugum Maxim |
| 25 | 7.1 | $[M-H]^-$ | 615.1735 | $597[M-H-H_2O]^-$，$477[M-H-C_7H_6O_3]^-$，$447[M-H-C_8H_8O_4]^-$，$431[M-H-C_8H_8O_5]^-$ | Iso-mudanpioside H | $C_{30}H_{32}O_{14}$ | Cortex Moutan |
| 26 | 7.27 | $[M-H]^-$ | 283.0621 | $268[M-H-CH_3]^-$，$240[M-H-CH_3-CO]^-$，$212[M-H-CH_3-CO-CO]^-$，$184[M-H-CH_3-CO-CO-CO]^-$ | Physcion | $C_{16}H_{12}O_5$ | Herba Selaginellae Moellendorffii |

| | | | | | | | |
|---|---|---|---|---|---|---|---|
| 27 | 7.35 | $[M+H]^+$ | 533.1285 | 285$[M+H-C_9H_{12}O_8]^+$，270$[M+H-C_9H_{12}O_8-CH_3]^+$，253$[M+H-C_9H_{12}O_8-CH_3-OH]^+$，225$[M+H-C_9H_{12}O_8-CH_3-OH-CO]^+$ | Calycosin-7-O-β-D-glucoside-6"-O-malonate | $C_{25}H_{24}O_{13}$ | Hedysarum multijugum Maxim |
| 28 | 7.45 | $[M-H]^-$ | 515.1196 | 353$[M-H-C_9H_6O_3]^-$，191$[M-H-2C_9H_6O_3]^-$，179$[M-H-C_9H_6O_3-C_7H_{10}O_5]^-$ | Dicaffeoylquinic acid | $C_{25}H_{24}O_{12}$ | Ecliptae Herba |
| 29 | 7.46 | $[M-H]^-$ | 685.2343 | 685$[M-H]^-$，299$[M-H-C_6H_{10}O_5-C_{11}H_{12}O_5]^-$ | Specnuezhenide | $C_{31}H_{42}O_{17}$ | Fructus Ligustri Lucidi |
| 30 | 7.66 | $[M-H]^-$ | 364.9971 | 285$[M-HSO_3]^-$，229$[M-HSO_3-2CO]$， | Luteolin sulfate | $C_{15}H_{10}O_9S$ | Ecliptae Herba |
| 31 | 7.73 | $[M-H]^-$ | 931.5247 | 799$[M-H-C_5H_8O_4]^-$， 637$[M-H-C_5H_8O_4-C_6H_{10}O_5]^-$，475$[M-H-C_5H_8O_4-C_6H_{10}O_5-C_6H_{10}O_5]^-$ | Saponins $R_1$ | $C_{47}H_{80}O_{18}$ | Panax notoginseng |
| 32 | 7.97 | $[M+H]^+$ | 489.1393 | 285$[M+H-C_8H_{12}O_6]^+$，270$[M+H-C_8H_{12}O_6-CH_3]^+$，225$[M+H-C_8H_{12}O_6-CH_3-OH-CO]^+$ | Calycosin-7-O-β-D-glucoside-6"-O-acetate | $C_{24}H_{24}O_{11}$ | Hedysarum multijugum Maxim |
| 33 | 8.23 | $[M+H]^+$ | 431.1317 | 269$[M+H-C_6H_{10}O_5]^+$，254$[M+H-C_6H_{10}O_5-CH_3]^+$，237$[M+H-C_6H_{10}O_5-OH-CH_3]^+$ | Ononin | $C_{22}H_{22}O_9$ | Hedysarum multijugum Maxim |
| 34 | 8.52 | $[M-H]^-$ | 599.1781 | 477$[M-H-C_7H_6O_2]^-$，137$[M-H-C_{23}H_{26}O_{10}]^-$，121$[M-H-C_{23}H_{26}O_{11}]^-$ | Mudanpioside C | $C_{30}H_{32}O_{13}$ | Cortex Moutan |
| 35 | 8.84 | $[M+H]^+$ | 285.077 | 270$[M+H-CH_3]^+$，253$[M+H-CH_3-OH]^+$，225$[M+H-CH_3-OH-CO]^+$， | Calycosin | $C_{16}H_{12}O_5$ | Hedysarum multijugum Maxim |
| 36 | 9.01 | $[M-H]^-$ | 267.066 | 267$[M-H]^+$，252$[M-H-CH_3]^+$，235$[M-H-CH_3-OH]^+$ | Formononetin | $C_{16}H_{12}O_4$ | Hedysarum multijugum Maxim |
| 37 | 9.01 | $[M+H]^+$ | 517.1354 | 269$[M+H-C_9H_{12}O_8]^+$，254$[M+H-C_9H_{12}O_8-CH_3]^{+,}$，237$[M+H-C_9H_{12}O_8-CH_3-OH]^+$ | Formononetin-7-O-β-D-glucopyranoside-6"-O-malonate | $C_{25}H_{24}O_{12}$ | Hedysarum multijugum Maxim |
| 38 | 9.61 | $[M-H]^-$ | 629.1858 | 583$[M-H-CH_2O_2]^-$，553$[M-H-C_2H_4O_3]^-$，535$[M-H-C_2H_60_4]^-$，431$[M-H-C_9H_{10}O_5]^-$ | Mudanpioside B | $C_{31}H_{34}O_{14}$ | Cortex Moutan |
| 39 | 9.64 | $[M-H]^-$ | 269.0458 | 251$[M-H-H_2O]^-$，241$[M-H-CO]^-$，225$[M-H-CO_2]^-$ | Apigenin | $C_{15}H_{10}O_5$ | Hedysarum multijugum Maxim |

| | | | | | | | |
|---|---|---|---|---|---|---|---|
| 40 | 9.66 | $[M-H]^-$ | 795.4507 | $471[M-H-C_5H_{10}O_5-C_7H_{10}O_5]^-$，$453[M-H-C_5H_{10}O_5-C_7H_{10}O_5-H_2O]^-$，$435[M-H-C_5H_{10}O_5-C_7H_{10}O_5-2H_2O]^-$，$417[M-H-C_5H_{10}O_5-C_7H_{10}O_5-3H_2O]^-$ | AstrasieversianinⅤ | $C_{42}H_{68}O_{14}$ | Hedysarum multijugum Maxim |
| 41 | 9.87 | $[M-H]^-$ | 299.0551 | $284[M-H-CH_3]^-$，$268[M-H-CH_3O]^-$ | Rhamnocitrin | $C_{16}H_{12}O_6$ | Hedysarum multijugum Maxim |
| 42 | 10.08 | $[M-H]^-$ | 1107.5913 | $945[M-H-C_6H_{10}O_5]^-$，$783[M-H-2C_6H_{10}O_5]^-$ $621[M-H-3C_6H_{10}O_5]^-$，$459[M-H-4C_6H_{10}O_5]^-$ | Ginsenoside $Rb_1$ | $C_{54}H_{92}O_{23}$ | Panax notoginseng |
| 43 | 10.46 | $[M+HCOO]^-$ | 829.4982 | $783[M-H]^-$,$604[M-H-C_6H_{11}O_6]^-$，$471[M-H-C_6H_{11}O_6-C_5H_9O_4]^-$，$453[M-H-C_6H_{11}O_6-C_5H_9O_4-H_2O]^-$，$435[M-H-C_6H_{11}O_6-C_5H_9O_4-2H_2O]^-$，$417[M-H-C_6H_{11}O_6-C_5H_9O_4-3H_2O]^-$ | Astragaloside | $C_{41}H_{68}O_{14}$ | Hedysarum multijugum Maxim |
| 44 | 11.25 | $[M+H]^+$ | 823.4095 | $647[M+H-C_6H_8O_6]^+$，$453[M+H-C_6H_8O_6-C_6H_{10}O_7]^+$ | Glycyrrhizinate | $C_{42}H_{62}O_{16}$ | Radix liquiritiae |
| 45 | 12.04 | $[M-H]^-$ | 941.512 | $471[M-H-C_5H_{10}O_5-C_{13}H_{20}O_9]^-$，$453[M-H-C_5H_{10}O_5-C_{13}H_{20}O_9-H_2O]^-$，$435[M-H-C_5H_{10}O_5-C_{13}H_{20}O_9-2H_2O]^-$，$417[M-H-C_5H_{10}O_5-C_{13}H_{20}O_9-3H_2O]^-$ | AstrasieversianinⅨ | $C_{48}H_{78}O_{18}$ | Hedysarum multijugum Maxim |
| 46 | 12.07 | $[M-H]^-$ | 941.5151 | $471[M-H-C_5H_{10}O_5-C_{13}H_{20}O_9]^-$，$453[M-H-C_5H_{10}O_5-C_{13}H_{20}O_9-H_2O]^-$，$435[M-H-C_5H_{10}O_5-C_{13}H_{20}O_9-2H_2O]^-$，$417[M-H-C_5H_{10}O_5-C_{13}H_{20}O_9-3H_2O]$ | AstrasieversianinⅪ | $C_{48}H_{78}O_{18}$ | Hedysarum multijugum Maxim |
| 47 | 12.31 | $[M-H]^-$ | 713.3552 | $713[M-H]^-$，$241[M-H-C_{30}H_{48}O_4]^-$ | Ecliander V | $C_{36}H_{58}O_{12}S$ | Ecliptae Herba |
| 48 | 13.85 | $[M-H]^-$ | 487.3435 | $469[M-H-H_2O]^-$，$425[M-H-H_2O-CO_2]^-$ | Tormentic acid | $C_{30}H_{48}O_5$ | Fructus Ligustri Lucidi |
| 49 | 14.3 | $[M-H]^-$ | 953.4713 | $471[M-H-C_6H_{11}O_6-C_{12}H_{15}O_9]^-$， | Malonylastragaloside I | $C_{48}H_{74}O_{19}$ | Hedysarum |

| | | | | | | | |
|---|---|---|---|---|---|---|---|
| | | | | 453[M-H-$C_6H_{11}O_6$-$C_{12}H_{15}O_9$-$H_2O$]$^-$，435[M-H-$C_6H_{11}O_6$-$C_{12}H_{15}O_9$-2$H_2O$]$^-$，417[M-H-$C_6H_{11}O_6$-$C_{12}H_{15}O_9$-3$H_2O$]$^-$ | | | multijugum Maxim |
| 50 | 17.61 | [M-H]$^-$ | 633.3817 | 615[M-H-$H_2O$]$^-$，589[M-H-$CO_2$]$^-$ | 3-O-cis-coumarin-mandelic acid | $C_{39}H_{54}O_7$ | Fructus Ligustri Lucidi |
| 51 | 17.84 | [M-H]$^-$ | 471.3472 | 453[M-H-$H_2O$]$^-$，427[M-H-$CO_2$]$^-$ | 2α-hydroxy-oleanolic acid | $C_{30}H_{48}O_4$ | Fructus Ligustri Lucidi |
| 52 | 21.4 | [M-H]$^-$ | 513.3575 | 495[M-H-$H_2O$]$^-$，469[M-H-$CO_2$]$^-$ | 19α-hydroxy-3-acetylbutyric acid | $C_{32}H_{50}O_5$ | Fructus Ligustri Lucidi |

**Verification of virtual screening results of network pharmacology**

**Effect of Zi Dian Fang on peripheral platelet count in ITP mice**

The effect of ZDF on peripheral platelet count in ITP mice is shown in Table S2. After 7 days of modeling, the platelet count in the model group was decreased compared with the control group (P < 0.01), while ZDF middle- and high-dose groups showed increased platelet numbers (P < 0.05). After modeling for 14 days, the platelet count of the model group was still decreased compared with the control group (P < 0.01). Mice in the ZDF middle-dose group showed increased numbers of peripheral platelets (P < 0.05). Thus, whereas the number of platelets in the model group remained depressed during the study's duration, ZDF treatment promoted platelet proliferation and elevated platelet counts.

Table S2 Effect of ZDF on peripheral platelets in ITP mice( $\bar{x} \pm s$， $10^9$/L)

| Grouping | Platelet count | |
|---|---|---|
| | Modeling after 7 days | Modeling after 14 days |
| Control group | 1051.0±158.9 | 1287.0±125.3 |
| Model group | 546.0±232.9$^{\#\#}$ | 790.6±109.3$^{\#\#}$ |
| ZDF high dose group | 713.7±149.5 | 857.6±161.9 |
| ZDF middle dose group | 884.9±258.5* | 1021.0±209.8** |
| ZDF low dose group | 832.7±244.9* | 938.0±199.2 |
| Prednisone acetate group | 906.5±227.3** | 1094.0±117.2** |

Note: Compared with the control group$^{\#\#}$P<0.01，$^{\#}$P<0.05；Compare with model group**P<0.01，*P<0.05

**Effect of Zi Dian Fang on the spleen index of ITP mice**

The effect of DZF on the spleen index of ITP mice is shown in Table S3. Compared with the control group, the spleen index was increased in the model group (P < 0.01), and this effect was attenuated in the ZDF low- and middle-dose treatment groups (P < 0.05). Thus, ZDF treatment effectively decreased splenomegaly in ITP mice.

Table S3 Effect of ZDF on spleen index of ITP mice( $\bar{x}$ ±s， n=10)

| Grouping | Spleen index（g/100g） |
|---|---|
| Control group | 0.6677±0.0702 |
| Model group | 1.1962±0.2509$^{\#\#}$ |
| ZDF high dose group | 1.0338±0.1189 |
| ZDF middle dose group | 0.9430±0.1843* |
| ZDF low dose group | 0.9683±0.1359* |
| Prednisone acetate group | 0.4909±0.08543** |

Note: Compared with the control group$^{\#\#}$P<0.01，$^{\#}$P<0.05；Compare with model group**P<0.01，*P<0.05.

**Effect of Zi Dian Fang on bone marrow megakaryocyte count in ITP mice**

The effect of ZDF on megakaryocyte count in ITP mice is shown in Table S4.

Compared with the control group, the total number of bone marrow megakaryocytes in the model group was increased (P < 0.05), and this effect was prevented in ZDF low-, middle- and high-dose animals. (P < 0.05). Meanwhile, a similar reduction was observed in the prednisone acetate group. Based on morphological classification, compared with the control group the number of granular megakaryocytes was increased, while that of thrombogenic megakaryocytes was decreased, in the model group (P < 0.05). Compared with the model group, the number of thrombogenic megakaryocytes was increased in the ZDF low-, middle-, and high-dose groups (P < 0.05). The latter results were similar to those observed in the prednisone acetate group.

Table S4 Effect of ZDF on bone marrow megakaryocytes in ITP mice( $\bar{x}$ ±s， n=10)

| Grouping | Total number of bone marrow megakaryocytes | Megakaryocyte classification(%) | | | |
|---|---|---|---|---|---|
| | | Promegakaryocytes | Granule megakaryocytes | Thrombocytogenous megakaryocytes | Bare nuclear megakaryocytes |
| Control group | 217.0±97.1 | 20.3±3.9 | 48.3±4.9 | 24.2±3.9 | 7.9±2.2 |
| Model group | 558.5±149.4$^{\#\#}$ | 23.5±3.4 | 54.2±5.8$^{\#}$ | 14.4±4.2$^{\#\#}$ | 7.6±1.4 |
| ZDF high dose group | 335.5±31.6* | 23.8±3.7 | 48.1±1.6* | 21.1±3.6** | 7.9±2.0 |
| ZDF middle dose group | 412.5±119.5 | 25.3±4.8 | 44.3±4.5* | 22.2±4.0** | 8.8±5.7 |
| ZDF low dose group | 493.5±124.7 | 21.4±3.9 | 47.9±4.3* | 20.7±4.9* | 9.7±0.8 |
| Prednisone acetate group | 336.6±81.9** | 21.6±3.6 | 44.3±4.7** | 23.9±2.0** | 10.1±1.8 |

Note: Compared with the control group $^{\#\#}$P<0.01，$^{\#}$P<0.05；Compare with model group **P<0.01 *P<0.05.

**Metabonomics analysis**

**Screening of differential metabolites**

Using the UPLC-Q-TOF/MS analysis technique, metabolomics analysis was performed on the different groups of serum samples. The BPI of the QC sample in the serum was shown in Figure S2.

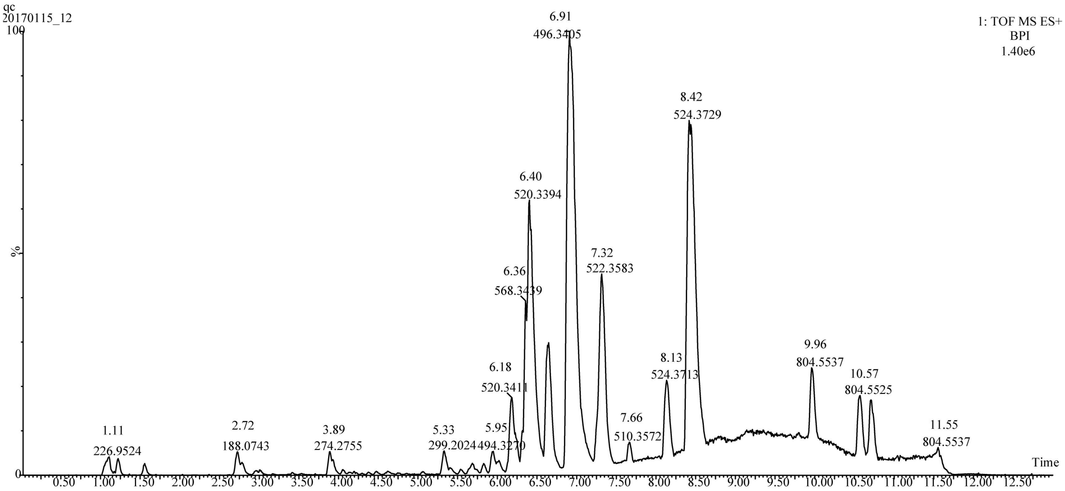


Figure S2 The base peak chromatogram (BPC) of QC sample in positive ion mode.

**Methodological study**

Table S5 Methodological study results

| Experimental name | RSD(peak area) | RSD(retention time) |
|---|---|---|
| Instrument precision | <11.4% | <1.6% |
| Method precision | <6.4% | <0.5% |
| Sample stability | <10.9% | <1.5% |
